# Food-induced cortisol secretion is comparable in lean and obese male subjects

**DOI:** 10.1530/EC-23-0126

**Published:** 2023-06-21

**Authors:** Patricia Arroyo Tardio, Gabriela Baldini, Eleonora Seelig

**Affiliations:** 1 University Hospital Basel, Basel, Switzerland; 2 University Clinic of Medicine, Cantonal Hospital Baselland, Liestal, Switzerland

**Keywords:** food, cortisol, lean, obese

## Abstract

**Objective:**

Hypercortisolism is a risk factor for obesity. Cortisol increases in response to food intake in lean subjects. In obese subjects, disturbances of the food-induced cortisol peak were reported, but data from sufficiently powered and well-controlled trials are lacking. Understanding the cortisol response to food is essential as amplified or recurrent cortisol surges could lead to hypercortisolism and contribute to obesity. Therefore, we investigate the cortisol response to food in lean and obese subjects.

**Design:**

This is a non-randomized, open-label study.

**Methods:**

We assessed serum cortisol values after a high-calorie meal in lean and obese male subjects. Cortisol levels were frequently assessed before and for 3 h after food intake.

**Results:**

A total of 36 subjects (18 lean and 18 obese) were included. There was no difference in overall cortisol levels between both groups during the study (area under the curve (AUC) obese: 55,409 ± 16,994, lean: 60,334 ± 18,001, *P* = 0.4). Total cortisol levels reached peak concentrations 20 min after food intake in both groups; the maximum cortisol increase was similar in both groups (cortisol increase obese: 69.6 ± 135.5 nmol/L, lean: 134.7 ± 99.7 nmol/L; *P* = 0.1). There was no correlation between body mass index and baseline cortisol values (*R*^2^ = 0.001, *P* = 0.83), cortisol increase (*R*^2^ = 0.05, *P* = 0.17), or cortisol AUC (*R*^2^ = 0.03, *P* = 0.28).

**Conclusions:**

This study demonstrates that high-calorie food intake causes an immediate and substantial cortisol response in lean and obese subjects and is independent of body weight.

**Significance statement:**

This study demonstrates that high-calorie food intake causes an immediate and substantial cortisol response in lean and obese subjects, independent of body weight. In contrast to the current literature, our findings show that the physiological cortisol response to food is intact in obesity. The substantial and prolonged increase further supports the hypothesis that frequent high-calorie meals cause hypercortisolism and aggravate weight gain.

## Introduction

The hypothalamopituitary–adrenal (HPA) axis tightly regulates cortisol secretion (1). Cortisol is secreted in a circadian rhythm with a brisk increase upon awakening and a nadir around midnight (1). Food is an external factor that modulates this circadian cortisol rhythm. The cortisol peak in response to food intake was first described in the 1970s and has been repeatedly reproduced in normal-weight subjects (2, 3, 4, 5). In lean populations, cortisol levels rise substantially and remain elevated for over 1 h after consuming a meal (4, 5). So far, few studies have investigated the food-induced cortisol peak in obesity, and the findings are heterogeneous. While two studies reported an enhanced cortisol increase, another study found a poor cortisol response after food intake (6, 7, 8). However, these studies were conducted in small populations or outpatient settings. Results from a well-controlled, sufficiently powered trial are lacking. Understanding the cortisol response to food is essential as amplified or recurrent cortisol surges could lead to hypercortisolism and contribute to obesity.

The consequences of hypercortisolism on weight are evident in patients with Cushing’s disease who have excessive endogenous cortisol secretion and abdominal weight gain (1). In common obesity, cortisol dysregulation is also observed, but changes are very subtle (9). However, even minor disturbances of the cortisol rhythmicity can have a disease-causing effect and potentially contribute to the development of obesity (1). The cause of cortisol dysregulation in obesity is still debated. Internal factors such as overexcitability of the HPA axis or differential tissue regulation are discussed (9). Whether meal-induced cortisol peaks may also play a role is not known. An indication that food is an external disruptor of cortisol secretion comes from a study of obese women where frequent consumption of high-calorie food was associated with elevated cortisol levels (10). However, whether obesity enhances the cortisol response to food and promotes hypercortisolism is unclear. We, therefore, investigated the cortisol response to a high-calorie meal in lean and obese subjects.

## Materials and methods

### Study design and population

We included 18 lean (body mass index (BMI) 18.5–25 kg/m^2^) and 18 obese (BMI >30 kg/m^2^) male participants in a nonrandomized, open-label study. All participants were between 18 and 40 years old. Subjects with clinically significant concomitant diseases, regular alcohol consumption (>30 g/day), and regular fitness training (>4 h per week) were excluded from the study.

Participants were recruited from the general population using flyers posted in the electronic and print media and from the obesity outpatient clinic of University Hospital Basel. Written consent was obtained from each participant after a full explanation of the purpose and nature of all procedures used. The study was conducted at the University Hospital Basel, Basel, Switzerland, between May 2020 and February 2021. The Ethics Commission of Northwestern and Central Switzerland (EKNZ) provided ethics approval, and the study was registered on https://clinicaltrials.gov/ (NCT04482738). The study was conducted in accordance with the ethical guidelines of the Declaration of Helsinki.

### Intervention

Participants were asked to refrain from strenuous physical activity and alcohol intake for 24 h and to remain fasted for at least 10 h before the study visit. Participants were admitted to the Clinical Trials Unit, University Hospital Basel, at 08:00 h.

After arrival, stress levels were assessed with the perceived stress questionnaire (11). Basal metabolic rate was measured with indirect calorimetry (Quark RMR, COSMED). Body composition was assessed with bioelectrical impedance analysis, which correlates well with dual-energy x-ray absorptiometry under standardized conditions (12). Blood sampling was initiated at least 1 h after the arrival at the research facility to prevent overlap with the cortisol awakening response, which occurs within 45 min of waking up (13, 14). An intravenous cannula was placed in the forearm 30 min before the first fasting blood sample was obtained at 09:30 h (±10 min). Then, participants were asked to consume a study meal containing 2080 kcal within 15 min. The study meal consisted of two buns, 162 g of cheese, two hard-boiled eggs, and two chocolate-flavored protein milkshakes, each with 40 g of added sugar (40% carbohydrates, 35% fat, and 25% protein).

After consuming the meal, blood samples were drawn every 10 min during the first hour and every 20 min during the following 2 h. Energy expenditure was remeasured 60 min after meal intake. Diet-induced energy expenditure was calculated as the difference between basal metabolic rate and energy expenditure at 60 min.

Serum total cortisol levels were measured with the commercial immunoassay Elecsys Cortisol II (Roche Diagnostics) at the laboratory of University Hospital Basel. Maximum cortisol increase was calculated as peak value minus the baseline value. The area under the curve (AUC) was calculated with the trapezoid rule based on all cortisol values over 180 min. Serum insulin was assessed with an immunoassay (Insulin ELISA, Mercodia, Uppsala, Sweden). Plasma glucose was measured with an enzymatic assay (Cobas modular analyzer, Roche Diagnostics). The HOMA index was calculated according to Matthews *et al.* (15). Thyroid-stimulating hormone (TSH) was measured with an immunoassay (Elecsys TSH, Cobas, Roche Diagnostics). The measurements were performed at the laboratory of University Hospital Basel.

Continuous data are expressed as mean (s.d.). Comparability between groups was analyzed by an unpaired *t*-test. A paired *t*-test was used for within-subject comparisons. Correlation analyses were performed using Pearson correlation. A *P*-value below 0.05 was considered significant. Statistical analyses were performed with GraphPad Prism v.9 (GraphPad Software).

## Results

Forty-three subjects were screened, and three were excluded after the screening due to BMI outside the inclusion range, cannabis consumption, or voluntary withdrawal before the study visit. Forty male participants were enrolled in the study; 4 participants dropped out due to difficulties with the insertion of the i.v. cannula and 36 completed the study (18 lean and 18 obese subjects). Obese males were slightly older compared to lean participants (Table 1). BMI and waist circumference were higher in obese subjects (Table 1). Fat and lean mass was significantly higher in obese compared to lean subjects (Table 1). There was no difference in systolic and diastolic blood pressure between the groups (Table 1).

**Table 1:** Baseline characteristics and results. Data are presented as mean ± s.d.

	Lean subjects (*n* = 18)	Obese subjects (*n* = 18)	*P*-value
Age (years)	24.1 ± 5.3	28.4 ± 5.8	0.03
Height (cm)	180.3 ± 6.5	178.9 ± 7.0	0.52
Weight (kg)	72.8 ± 7.6	113.8 ± 14.5	<0.001
BMI (kg/m^2^)	22.3 ± 1.3	35.6 ± 4.7	<0.001
Waist circumference (cm)	87.9 ± 5.7	120.6 ± 13.8	<0.001
Systolic blood pressure (mm Hg)	133.9 ± 11.3	138.4 ± 13.2	0.29
Diastolic blood pressure (mm Hg)	76.7 ± 8.8	83.7 ± 11.6	0.06
Fat mass (kg)	10.7 ± 4.0	36.6 ±11.1	<0.001
Lean mass (kg)	62.1 ± 6.6	77.1 ± 6.8	<0.001
Resting energy expenditure (kcal/24 h)	1936 ± 328	2325 ± 465	0.007
Diet-induced energy expenditure (kcal/24 h)	779 ± 405	896 ± 385	0.40
Cortisol baseline (nmol/L)	340 ± 107	349 ± 170	0.85
Cortisol peak (nmol/L)	475 ± 124	419 ± 125	0.18
Maximum cortisol increase (nmol/L)	134.7 ± 99.7	69.6 ± 135.5	0.10
Cortisol 3 h AUC	60,334 ± 18001	55,409 ± 16994	0.40

AUC, area under the curve; BMI, body mass index.

Resting energy expenditure was higher in obese subjects (Table 1). Diet-induced thermogenesis was comparable in both groups (Table 1). Both groups’ stress levels were moderate and similar (lean: 33 ± 4 points, obese: 32 ± 4 points, *P* = 0.3).

**Figure 1:**
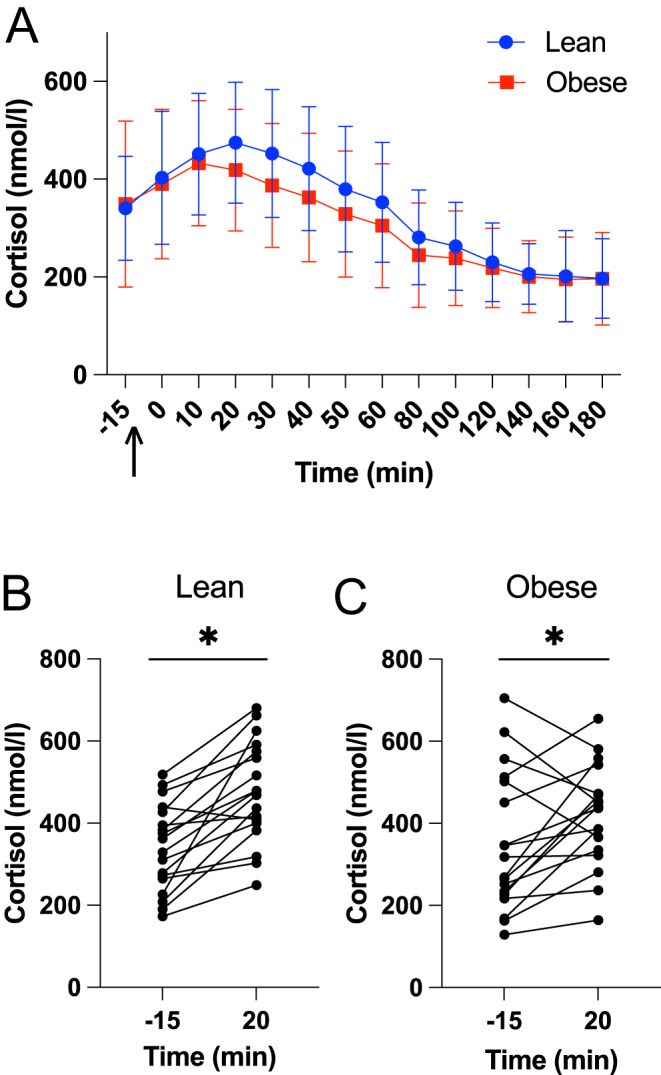
Panel (A) shows mean cortisol values for lean (blue) and obese (red) subjects throughout the trial; the arrow indicates the time of food consumption. Error bars indicate the s.d. Panels (B) and (C) show the increase from baseline to peak cortisol values at 20 min for lean (*P* < 0.001) and obese (*P* = 0.04) subjects. Differences were evaluated with a paired *t*-test.

Baseline cortisol levels before meal intake were similar in both groups (Table 1, Fig. 1A). After the meal, both groups had a significant cortisol increase, reaching peak levels at 20 min (change from baseline to 20 min in lean subjects: 135 ± 99 mmol/L, *P* < 0.001; obese: 70 ± 135 mmol/L, *P* = 0.04; Fig. 1B and C). Total cortisol levels reached similar peak concentrations around 20 min after food intake (Table 1, Fig. 1A). There was no difference in maximum cortisol increase nor cortisol levels between the two groups for the total duration of the study (Table 1, Fig. 1A). There was no correlation between BMI with baseline cortisol values (*R*^2^ = 0.001, *P* = 0.83), maximum cortisol increase (*R*^2^ = 0.05, *P* = 0.17), or cortisol AUC (*R*^2^ = 0.03, *P* = 0.28). Cortisol levels of four obese and one lean participant did not increase after meal intake. All non-responders had baseline cortisol levels in the upper third range (Fig. 1B and C). The lean non-responder had the highest stress level of all study participants (41 points). The four obese non-responders did not stand out regarding stress levels (30.7 ± 5.6), age (29.5 ± 7.3), BMI (33.3 ± 3.7), HOMA index (3.1 ± 1.7), or TSH (1.7 ± 0.9).

Glucose levels at baseline were comparable between groups (lean: 5.2 ± 0.7 mmol/L, obese: 5.4 ± 0.8 mmol/L, *P* = 0.42). Insulin levels were higher in obese subjects (lean: 22 ± 18 pmol/L, obese: 80 ± 49 pmol/L, *P* < 0.001). Accordingly, the HOMA index was elevated in obese subjects (lean: 0.9 ± 0.8; obese: 3.1 ± 1.9, *P* < 0.001). There was no correlation between the HOMA index with baseline cortisol values (*R*^2^ = 0.01, *P* = 0.49), maximum cortisol increase (*R*^2^ = 0.09, *P* = 0.06), or cortisol AUC (*R*^2^ = 0.008, *P* = 0.59).

TSH values were within the normal range for both groups (lean: 1.6 ± 0.7; obese 2.3 ± 1.4). Within this normal range, TSH levels in obese were higher compared to lean subjects (*P* = 0.007). Two obese subjects had minimally elevated TSH levels (0.5 and 0.3 mIU/L above the normal upper range) without experiencing symptoms of hypothyroidism.

## Discussion

Here, we show that obese and lean subjects have a substantial cortisol increase in response to food. We find that consuming a high-calorie meal leads to a cortisol surge of around 100 nmol/L, independent of body weight. Furthermore, cortisol levels remain elevated for at least 1 h before returning to baseline values in both groups. These findings align with earlier studies in lean subjects, where cortisol levels increased around 90 nmol/L and remained elevated for a prolonged period (5). As mild chronic cortisol excess is associated with cardiovascular diseases (16), recurrent cortisol surges of the observed magnitude and duration could have detrimental metabolic effects.

So far, studies on meal-induced cortisol in obese subjects were scarce, and results were heterogeneous. In an observational study, obesity was associated with a poor food-induced cortisol increase in men with little circadian variation (6). Still, in stress-sensitive men with high cortisol variation, elevated cortisol levels after lunch were also associated with obesity. This study relied on self-measurements of salivary cortisol levels after a standardized lunch at home. An inpatient trial in obese and lean children found that the cortisol peak was similar after lunch; however, obese children had a more substantial cortisol rise after breakfast (7). Again, cortisol was measured in saliva, which is more error prone after the consumption of a meal due to blood leakage or dilution. An enhanced cortisol response in obese subjects was reported in a small study in adults (8). In this study, cortisol was lower in obese subjects before the meal but reached similar peak values after food intake. Other external activators of the HAP axis, such as stress, were not monitored. Thus, it remains challenging to interpret the difference in baseline cortisol. In our study, cortisol levels at baseline were comparable in both groups; furthermore, there was no correlation between baseline cortisol and BMI, indicating that baseline cortisol levels are independent of body weight. We also found that both groups' stress levels were comparable and moderate and therefore were able to exclude stress as a potential bias. Indeed, the lean study participant with the highest stress level had a high baseline cortisol and no postprandial cortisol increase; thus, the HPA axis was already stimulated before the meal, indicating the importance of evaluating stress in this setting. Still, four obese subjects who did not respond to the meal also had high baseline cortisol, but stress levels were moderate, and no other characteristics set them apart. While the cause of the HPA axis stimulation in these subjects remains unknown, it shows how a recent cortisol pulse can render the HPA axis less responsive to a new stimulus (1).

While we show that body weight does not influence the food-induced cortisol response, other factors such as the number of calories, the macronutrient content, and the meal's timing might be important.

Here, we tested the response to a high-calorie meal, while most studies used meals with fewer calories. Although the observed cortisol peaks are comparable to other studies, we find that the peak is reached much earlier (<20 min compared to <60 min), pointing to a prolonged cortisol elevation with high-calorie meals (4, 5, 8). Indeed, a few high-calorie meals impact cortisol levels more than numerous smaller meals, despite containing the same total amount of calories (17).

The macronutrient component that causes food-induced cortisol rise has also been investigated. Earlier studies suggested that proteins are the primary driver (4, 18). A more recent study convincingly shows that all three major macronutrient components contribute to the postprandial cortisol peak to a similar extent (5). However, the source of circulating cortisol seems to differ between the macronutrient components. Protein and fat primarily induce adrenal cortisol production; carbohydrates cause substantial enzymatic cortisol regeneration in the liver by 11beta dehydrogenase 1 (11beta HSD1). As the enzyme activity of 11beta HSD1 is upregulated in obesity, high carbohydrate meals may cause higher circulating cortisol concentrations in obese subjects (19). We used a mixed meal reflecting real-life conditions (40% carbohydrates, 35% fat, and 25% protein). Whether meals with different macronutrient ratios cause different cortisol increases in obese compared to lean subjects remains to be investigated. Furthermore, in-depth studies with mass spectrometry analysis and isotope tracers will be needed to analyze the source of the circulating cortisol in obese subjects.

The meal timing is another factor that impacts the food-induced cortisol peak. Following a circadian rhythm, cortisol reaches its highest concentrations within 45 min of waking up (cortisol awakening response) and lowest at midnight (13, 14). Here we prevented overlap with the cortisol awakening response by delaying the initiation of blood sampling for more than 1 h to mid-morning. Interestingly, meal intake at lunchtime produces higher cortisol peaks than in the evening (2). Still, already small cortisol surges after a late-night dinner can have a negative metabolic impact by causing nocturnal glucose intolerance (20). Moreover, as late-night snacking is prevalent among obese subjects, misalignment of circadian rhythm by frequent cortisol pulses may contribute to metabolic deterioration (21). In addition, late-night salivary cortisol has been recommended as a valid diagnostic method for Cushing’s disease (22). However, a postprandial increase in cortisol in the late evening could lead to false-positive results and, therefore, late-night salivary cortisol may lack diagnostic accuracy for Cushing’s disease.

A limitation of our study is the exclusion of women, as hormone fluctuations may alter cortisol levels. Furthermore, obese subjects were slightly older compared to normal-weight participants, but all participants were in or around their twenties. Therefore, we do not expect an effect of age on cortisol levels. Still, cortisol secretion increases with age, and only young men were recruited for the study (23). Thus, our study findings cannot be generalized to the overall population. Before study inclusion, the participants were not biochemically tested for adrenal insufficiency or hypercortisolism, as the risk for such a disease was considered negligible in this young and healthy population (24, 25). Mean TSH levels were within the normal range for both groups but overall higher in obese subjects. While hypothyroidism can affect body weight by decreasing energy expenditure, we did not observe an impact on basal metabolic rate in obese subjects.

In conclusion, we show an immediate and pronounced cortisol response to food independent of body weight. However, whether frequent high-calorie food intake causes mild hypercortisolism and contributes to the development of obesity remains to be investigated in further studies.

## Declaration of interest

The authors declare no conflict of interest.

## Funding

This work was supported by the Research Fund, University Basel awarded to E.S.
